# Seroprevalence of anti-SARS-CoV-2 IgG antibodies among vaccinated healthcare workers in Bamako, Mali

**DOI:** 10.4314/ahs.v25i3.4

**Published:** 2025-09

**Authors:** Etienne Dembele, Yacouba Cissoko, Anou M Somboro, Aichata Dembele, Josue Togo, Djibril M Bah, Issiaka Camara, Ousmane Kodio, Mohamed Tolofoudie, Bourahima Kone, Antieme CG Togo, Bocar Baya, Bassirou Diarra, Yeya Dit Sadio Sarro, Robert L Murphy, Almoustapha I Maiga, Jane L Holl, Mamoudou Maiga, Sounkalo Dao

**Affiliations:** 1 Centers for Disease Control (CDC) Georgia, USA; 2 Institute for Global Health, Northwestern University, Chicago, Illinois, USA; 3 University Clinical Research Center (UCRC) Laboratory, University of Sciences, Techniques and Technologies of Bamako (USTTB), Bamako, Mali; 4 Antimicrobial Research Unit, College of Health Sciences, University of KwaZulu-Natal, Durban 4001, South Africa; 5 Department of Infectious Diseases and Tropical Medicine, Point “G” University Teaching Hospital, Bamako, Mali; 6 Department of Public Health Sciences, Penn State College of Medicine, Hershey, Pennsylvania, USA; 7 Centers for Disease Control (CDC) Bamako, Mali; 8 Department of Neurology and Center for Healthcare Delivery Science and Innovation, University of Chicago, Chicago, Illinois, USA

**Keywords:** COVID-19 vaccines, SARS-CoV-2 seroprevalence, healthcare workers, Bamako, Mali

## Abstract

**Introduction:**

Like many other countries, Mali, a West African country, has encountered various obstacles in the fight against the transmission of severe acute respiratory syndrome coronavirus 2 (SARS-CoV-2). Despite resource constraints, however, the country implemented containment strategies. Therefore, in early 2021, Mali initiated a vaccination campaign as a tangible defense against COVID-19, prioritizing the administration of the first vaccine doses to healthcare personnel. Consequently, we found assessing anti-SARS-CoV-2 IgG antibody levels important to gauge the efficacy of vaccines administered to frontline healthcare workers in Bamako, Mali.

**Methods:**

The study enrolled 172 vaccinated front-line healthcare workers from referral hospitals in Bamako, Mali, between March and June 2022. Serum samples were subjected to enzyme-linked immunosorbent assay (ELISA) to assess the levels of anti-SARS-CoV-2 IgG antibodies. Prevaccination serum samples served as controls. Chi-square and Mann-Whitney tests were used to compare proportions and means.

**Results:**

Among the 172 participants, 98.2% had high levels of anti-SARS-CoV-2 spike protein antibodies; only 1.6% (n=2) were seronegative. The majority, 62.2% (n=107), received a two-dose vaccination schedule, and the Astra Zeneca® vaccine was the most widely used (52.3%). The average level of postvaccine antibodies was significantly greater in participants who received two doses of vaccine than in those who received one dose (33.7 index vs. 29.1 index; p=0.02).

**Conclusions:**

Most healthcare workers exhibited favorable vaccine responses, as indicated by their positive reactivity to anti-SARS-CoV-2 IgG spike proteins. The nature and dosage of the vaccines influenced the antibody response, with a notable advantage observed for individuals who received a two-dose regimen. These findings underscore the importance of continuous research and evaluation to understand and enhance vaccine effectiveness.

## Introduction

In late 2019, a respiratory infection caused by a newly emerged virus first identified in Wuhan (China) began to spread worldwide, causing a pandemic^1^–^3^. The genetic sequencing of the virus indicates that it is a beta coronavirus closely related to severe acute respiratory syndrome (SARS) virus, hence its name, SARS-CoV-2^4^.

Coronaviruses are RNA viruses of the family Coronaviridae responsible for digestive and respiratory infections in humans and animals^5^. SARS-CoV-2 is transmitted from one infected person to another through respiratory droplets and aerosols when an infected person breathes, coughs, sneezes, screams, or speaks^6^,^7^. SARS-CoV-2 is also transmitted through contact with a hand soiled from surfaces contaminated by the nasal secretions of an infected person with mucous membranes on the face.

Early in the start of the pandemic, most developed nations adopted a wide range of measures (e.g., handwashing, masking, social distancing, and closures) to reduce the transmission and spread of the infection. Some African nations quickly adopted similar measures. Mali, a low- and middle-income country in West Africa with approximately 22 million people and a very limited resource health system, has adopted many of these measures, although the feasibility of these measures is questionable given the lack of resources (e.g., masks) and the precarious economic situation (e.g., closures) of many inhabitants. The first cases of COVID-19 in Mali were detected in March 2020, yet by the end of 2022, only 33,000 cases and 742 deaths were reported^8^. However, a 2020 report showed that front-line healthcare workers are at greater risk for reporting a positive COVID-19 test than the general community (adjusted HR 11.61, 95% CI 10.93-12), and in an initial study, we assessed the SARS-CoV-2 seroprevalence of unvaccinated front-line healthcare workers and showed a continuous increase in seropositivity, from 50% to 70%, between November 2020 and June 2021 9.

Early in 2021, Mali undertook a vaccination campaign against COVID-19. Among the first people to receive this vaccination were healthcare workers, not only because they were at high risk of contracting the disease but also because they wanted to provide an example for the population to adhere to the vaccination program. Many questions have been raised about the efficacy and safety of vaccines to protect against COVID-19, which partly explains the reluctance of most people to adhere to vaccination. In addition, no report has evaluated the impact of the various COVID-19 vaccines administered to the Malian population. Therefore, in this study, we assessed the level of anti-SARS-CoV-2 IgG antibodies induced after vaccination to understand the impact of the various vaccines available in Mali on protecting healthcare workers against COVID-19.

## Methods

### Study Design and Population

This cross-sectional study was conducted among frontline healthcare workers (e.g., nurses, doctors, and laboratory technicians). Healthcare workers at the four main public hospitals in Bamako, Mali (Hospital of Point-G, Hospital of Gabriel Touré, Hospital of Kati, and Hospital of Mali) who had been vaccinated against COVID-19 between March and June 2022 were included in this study. All participants provided IRB-approved informed consent before any study procedures were undertaken.

### Study Procedures

**Clinical Procedures:** Participants responded to a questionnaire administered by a trained research assistant that collected information about their sociodemographic characteristics (age, sex, occupation); prior signs/symptoms of COVID-19 infection (signs, symptoms, type of sample tested, type of test, result); comorbidities (hypertension, diabetes, renal failure, cardiac, HIV, hepatitis); individual level of protection practice (e.g., PPE use, hand washing, distancing); and SARS-CoV-2 vaccination details (type(s) of vaccine, date(s) of vaccination, and number of doses received). A physical examination was performed, focusing on physical signs suggestive of COVID-19. Vital signs (temperature, blood pressure, heart rate, respiratory rate, oxygen saturation, weight, and height) were obtained. At least 5 ml of blood was collected via venipuncture in a serum-separating tube (SST). COVID-19-related symptoms in the past three months were recorded.

**Laboratory Procedures:** All collected blood samples were centrifuged within two hours of collection, and the serum was aliquoted into cryotubes for storage at -20°C prior to analysis. The serum samples were then transferred to the Immunology Core Laboratory of the University Research Clinical Center (UCRC). A qualitative assay to detect Anti-SARS-CoV-2 spikeprotein IgG was conducted using an enzyme-linked immunosorbent assay (ELISA).

**SARS-CoV-2 IgG ELISA:** An enzyme-linked immunosorbent assay (SARS-CoV-2 IgG ELISA kit [ENZ-KIT170-0001]) was used to detect IgG antibodies against SARS-CoV-2 in human serum. The intensity of coloration proportional to the amount of anti-SARS-CoV-2 IgG antibodies in the serum of patients was tested through a colorimetric assay. The assay was conducted following the manufacturer's instructions. Briefly, the ELISA kit (ENZ-KIT170-0001) was brought to room temperature, and the serum was heat-inactivated at 60°C for 30 min. Then, the diluent reagent was used to dilute the serum samples at a 1:10 ratio. The diluted samples were loaded in a 96-well plate and incubated for 30 minutes at 37°C. The contents of the wells were aspirated and washed using an automated washing machine, and 100 µL of the HRP conjugate was added to the corresponding wells and incubated at 37°C for 15 minutes. Shortly thereafter, the wells were aspirated and washed, and then 100 µL of TMB substrate was added to each well and incubated in the same manner. This reaction was completed by adding 50 µL of stop solution. Within 15 minutes of adding the stop solution, the plate was read using a microplate reader (VersaMaxTM ELISA Microplate Reader).

The interpretation of the assay was adjusted and validated in our previous study^9^. According to the manufacturer's guidelines, an enzyme-linked immunosorbent assay (ELISA) optical density (OD) index greater than 1.1 is considered positive, an OD index between 0.9 and 1.1 is considered equivocal, and an OD index less than 0.9 is considered negative for anti-SRAS-CoV-2 IgG^10^,^11^. However, this did not apply to our study population with high background signals. Adjustments are generally suggested for serological testing in populations with high antibody/protein background signals and frequent infections, such as in Mali^10^,^11^. Therefore, we determined the Malian population OD index for this kit using serum samples collected well before COVID-19. We considered samples with an OD index < 3 as seronegative samples with an OD index of ≥ 3 but < 5 as equivocal samples and samples with an OD index > 5 as seropositive samples.

### Statistical Analysis

The collected data were analyzed using Excel 2016 and SPSS 16. SPSS was used to record the data, while Excel was used to clean and present the data using graphs and charts. The questionnaires were used to collect symptom and comorbidity data. The results are presented as participant numbers (n) and proportions (%). The chisquare test was used to compare proportions in the bivariate analysis. Mann-Whitney tests were used to compare outcomes between two independent groups. The significance threshold of the tests was at the 5% level.

### Ethical considerations

The informed consent document and study protocol were approved by the Ethics Committee (EC) of the University of Bamako, Faculty of Medicine, and Odon-to-Stomatology (Reference Number: No2021/134/CE/USTTB.

## Results

### Study Population and Clinical and Sociodemographic Data of the Participants

A total of 172 front-line healthcare worker participants vaccinated against SARS-CoV-2 were included in the study. Most participants were men (52.3%), with a sex ratio (M/F) of 1.09. The age of participants ranked from 18 to 65 years, with 83.1% (n=143) being in the age group of 18-40 years and 16.9% (n=29) being in group of 41-65 years old. Nurses and physicians were the most represented professions, 28.4% (n=49) and 16.9% (n=29), respectively. Of the 172 participants, 10.5% (n=18) reported comorbidities, with high blood pressure (hypertension) being the most reported (6, 3.5%). Headache and fever were the most commonly recorded clinical signs, 13.9% (n=24) and 9.3% (n=16), respectively (Table 1).

**Table 1:** Clinical and Socio-Demographics of the Participants

Characteristics	Participants

	n = 172
**Age Range in Years n (%)**	
[18-40]	143 (83.1)
[41-65]	29 (16.9)
Male, n (%)	90 (52.3)
Female, *n* (%)	82 (47.7)
**Occupation n (%)**	
Nurse	49 (28.4)
Physician	29 (16.9)
Janitor	25 (14.5)
Laboratory Technician	23 (13.4)
Hygienist	15 (8.7)
Others *	31 (18.0)
**Symptoms prior vaccination n (%)**	
Headache	24 (13.9)
Fever	16 (9.3)
Pain	11 (6.4)
Sore Throat	7 (4.1)
Dry cough	6 (3.5)
Rhinorrhea	6 (3.5)
Nausea	5 (2.9)
Asthenia	5 (2.9)
Productive cough	3 (1.7)
Shortness of Breath	3 (1.7)
Nasal congestion	3 (1.7)
Diarrhea	2 (1.2)
Chills	1 (0.6)
**Comorbidities n (%)**	
None	154 (89.0)
HTA	6 (3.5)
Obesity	5 (2.9)
Asthma	2 (1.2)
Diabetes	2 (1.2)
Other**	3 (1.7)

* *Receptionist, internship fellows, etc*….

** *Smoking, alcohol use*

### Types of vaccines used at the first dose

The most widely used COVID-19 vaccines for the first dose were Astra Zeneca, Johnson & Johnson 90 (52.3%) and 65 (37.8%). Three other vaccines (Covishield, Neuter, and Pfizer) were also used in Mali at the time of the study period but were not used frequently.

### Types of Vaccines Used at the Second Dose

All participants who received the first dose of AstraZeneca completed their vaccination by receiving a second dose of the same AstraZeneca vaccine. The same was done for the three other types of vaccines. However, those who received Johnson & Johnson did not receive a second dose, as one dose was deemed adequate during the study period.

### Time between the First and Second Doses

The average time between the first and second doses was 32.2 days (± 6.1), ranging from 15 to 56 days, with more than half of the participants 58.9% (n=63) receiving a second dose at least 30 days after the first dose. (Table 4).

**Table 4:** Time between the first and second doses

Time (days)	Number(n)	Percentage (%)
**[15 – 30]**	44	41.1
**[31 – 60]**	63	58.9
**Total**	107	100

### Immune Response After COVID-19 Vaccination (Antibody Levels)

Of the 172 participants, 98.2% were sero-positive with high levels of Anti-SARS-CoV-2 spikeprotein IgG (≥ 5 index); only 2 (1.6%) were sero-negative.

### Correlation of the Induced Levels of Anti-SARS-CoV-2 spikeProtein IgG with Age, Sex and History of COVID-19

**Figure 1:**
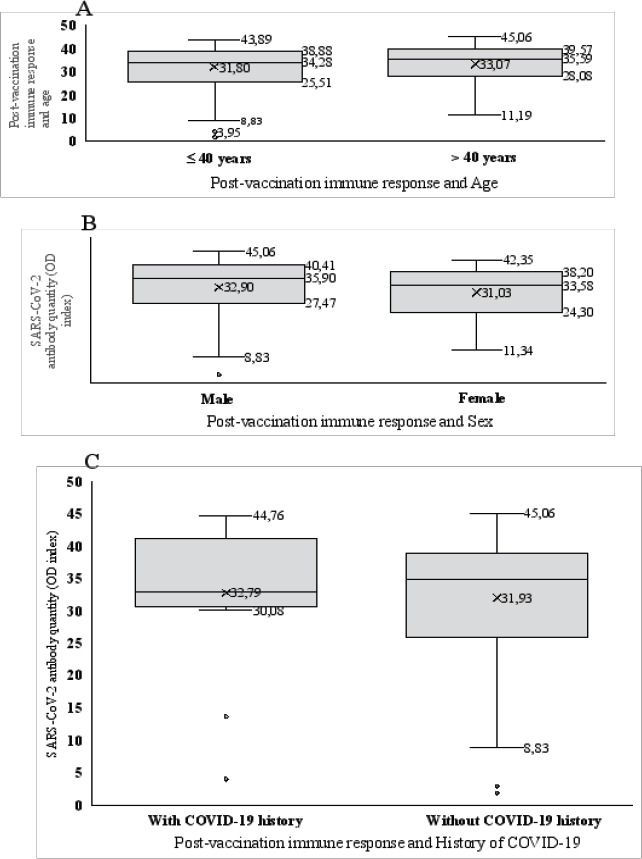
**(A)** Relationship between post-vaccination response and age, (**B**) Relationship between post-vaccination response and sex, and (**C**) Relationship between post-vaccination response and COVID-19 history

The average level of SARS-CoV-2 antibodies induced by COVID-19 vaccination was 31.8±9.15 in those ≤40 years old and 33.1±9.16 in those >40 years old. There was no significant difference in the level of the Anti-SARS-CoV-2 spikeprotein IgG between age groups (Mann-Whitney test: p=0.744) (Fig. 1A).

The average level of SARS-CoV-2 antibodies induced by COVID-19 vaccination was 32.9± 9.5 in males and 31.0± 8.6 in females, which was not a statistically significant difference (Mann-Whitney test: p=0.187) (Fig. 1B).

The average level of IgG antibodies induced by COVID-19 vaccination was 32.7±10.7 in participants with a history of COVID-19 and 31.9±9 in those who had no history of COVID-19; this difference was not statistically significant (Mann-Whitney test: p=0.716) (Fig. 1C).

### Expression Level of Anti-SARS-CoV-2 spikeProtein IgG After One Dose of Johnson & Johnson and Two Doses of Other Vaccines

**Figure 2:**
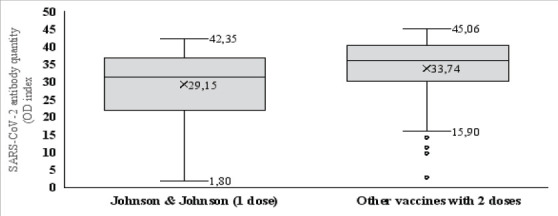
Relationship between postvaccination response and number of doses

The average postvaccination Anti-SARS-CoV-2 antibody levels after two doses were 33.7±8.2 and 29.1±9.8 in participants who received one dose of the Johnson & Johnson vaccine, respectively, which were statistically significant (Mann-Whitney test: p=0.02). (Fig. 2)

### The Expression Levels of Anti-SARS-CoV-2 spikeProtein IgG and the Time between the 1^st^ Dose and Sampling

**Figure 3:**
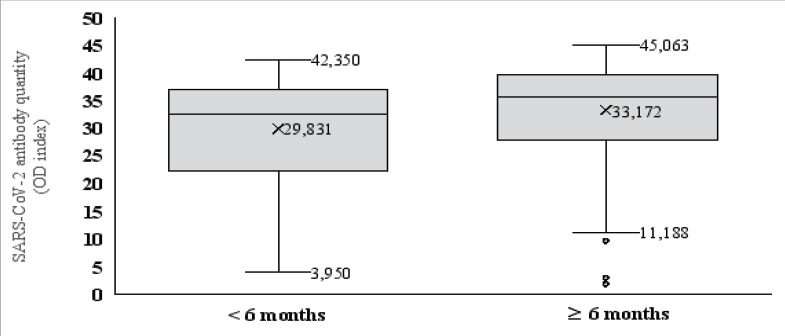
Relationship between postvaccination response and time between the 1st dose and sampling

The average postvaccine Anti-SARS-CoV-2 antibody level was 29.8±9.8 in participants who received their 1st dose of vaccine more than 6 months ago and 33.1±8.2 in participants who received their 1st dose less than 6 months ago, which was a statistically significant difference (Mann-Whitney test: p=0.023) (Fig. 3).

### The Expression Level of Anti-SARS-CoV-2 spikeProtein Ig and the Time between the 2^nd^ Dose and Collection

**Figure 4:**
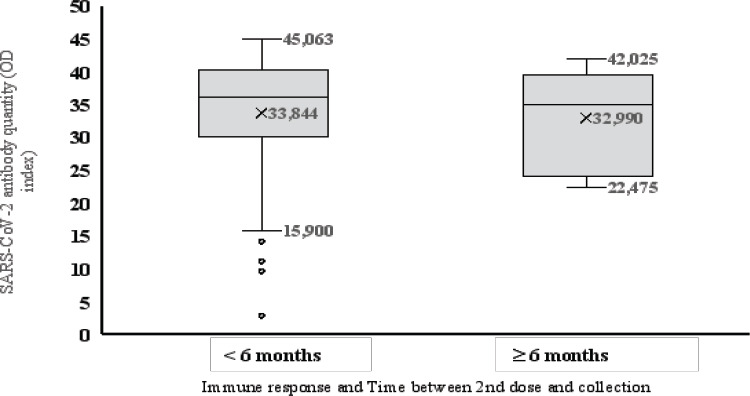
Relationship between postvaccination response and time between the second dose and sample collection

The average postvaccine Anti-SARS-CoV-2 antibody levels of participants who received a 2nd dose of vaccine <6 months after the 1st dose were 33±7.3 and 33.8±8.4, respectively, in participants who received a 2nd dose of vaccine >6 months after the 1st dose, and no statistically significant differences were observed (Mann-Whitney test: p=0.07). (Fig. 4)

### Comparison of the Expression Levels of Anti-SARS-CoV-2 spikeProtein IgG Before and After Vaccination

**Figure 5:**
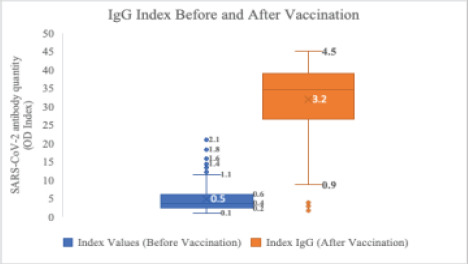
Comparison of IgG Indices Before and After Vaccination

Before vaccination, the anti-SARS-CoV-2 antibody IgG levels were derived from a previous study (Somboro et al.). The level of the Anti-SARS-CoV-2 spikeprotein IgG was considerably lower before vaccination than after vaccination, indicating that vaccination induced antibodies against COVID-19. (Fig. 5)

### Comparison of the Expression Levels of Anti-SARS-CoV-2 spikeProtein IgG by Type of Vaccine

**Figure 6:**
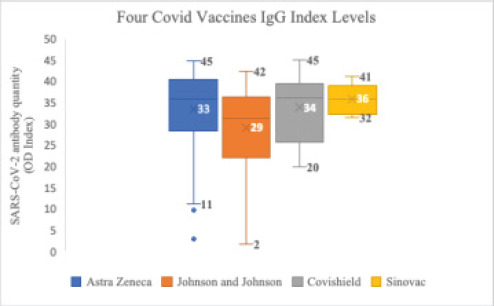
Comparison of the IgG indices of the four COVID-19 vaccines

The OD indices of three of the vaccines (Astra Zeneca, Covishield, and Sinovac) were similar. Johnson & Johnson showed a lower level of antibody than the three other vaccine types, but the difference was not significant (Fig. 6).

## Discussion

This study elucidated the Anti-Anti-SARS-CoV-2 antibody response after one and/or two doses of vaccines among front-line healthcare workers (HCWs) in four public hospitals in Bamako, Mali. The different vaccination protocols applied in Mali followed the WHO's initial recommendations on vaccination against COVID-19. The vaccines were introduced in Mali through the COVAX mechanism and within the framework of bilateral cooperation partners of Mali^12^. Johnson & Johnson®, Pfizer-BioNtech® and AstraZeneca® vaccines (Oxford and Covishield) are the main vaccines used in the WHO African Region, including Mali^13^. For the general population to adhere to the vaccination program, as health workers are at greater risk, the vaccination first involved people in frontline healthcare workers and political leaders. This prompted us to evaluate the type of vaccine used and at which frequencies the doses were delivered as well as to meaure their effectiveness through the quantification of produced Anti-SARS-CoV-2 antibody levels in the HCW population in Bamako, Mali.

Overall, all participants who had received the COVID-19 vaccine, including one or two doses, had a high rate of seropositivity for IgG anti-spike antibodies. SARS-CoV-2 IgG anti-spike antibodies are detected in individuals upon SARS-CoV-2 infection or vaccination and are assumed to protect against infection or reinfection. Herein, we observed that 98.2% of the study participants vaccinated against COVID-19 had a positive quantitative immune response (i.e., a significant level of anti-SARS-CoV-2 antibodies against the IgG spike protein (≥ 5 OD index). Several authors have also reported a high postvaccine Anti-SARS-CoV-2 antibody seropositivity rate in at least 97% of vaccinated HCWs^14^-^16^.

A comparative expression level of the anti-Anti-SARS-CoV-2 spike protein demonstrated a similar OD index value for three of the four vaccine types (Astra Zeneca, Covishield, and Sinovac) administered to the participants. In contrast, Johnson & Johnson showed a lower level of antibody response. Chanda Mog et al.^17^, in a similar study, showed that the COVID-19 vaccine covishield was associated with a higher rate of anti-SARS-CoV-2 IgG antibody seroprevalence, which correlates with our findings. However, we noticed a lower OD index value with the Johnson & Johnson vaccine, which may be explained by the single dose received, while the other vaccines were mostly administered more than a single dose.

We examined the correlation between the sociodemographic characteristics of the healthcare workers and the seropositivity rate in this study population. In terms of age, sex, and previous COVID-19 history, there were no significant differences in the level of anti-SARS-CoV-2 IgG antibodies. Indeed, the production of antibodies derived from T lymphocytes and the quantity of B lymphocytes decrease with age, and at the same time, the humoral response against infections and postvaccination may be insufficient^18^. Various studies have shown that the postvaccination humoral response to certain vaccinations, such as hepatitis A and B, tick-borne encephalitis, influenza, tetanus, and COVID-19, is age-proportional^19^-^22^. In our study, the average level of SARS-CoV-2 antibodies induced by COVID-19 vaccination was 31.8±9.15 in participants under 40 years of age and 33.1±9.16 in participants over 40 years of age, with no statistically significant difference (p=0.744). The quantitative production of anti-SARS-CoV-2 antibodies postvaccination is therefore not proportional to age, as observed in our study, and has also been demonstrated in a study conducted by Uysal et al.^15^. The study population was mostly male and relatively young, with an average age of 29 years. Studies conducted by Somboro et al.^9^ and Maiga et al.^23^ reported similar findings that healthcare workers in Mali are relatively young, with approximately 58% male and an average age of 28 to 32 years^9^, ^23^. The quantitative postvaccine production of SARS-CoV-2 antibodies was not correlated with sex (p=0.187) or previous COVID-19 infection (p=0.716). Nevertheless, Bayram et al found a significantly greater antibody level in health workers with a history of COVID-19 who received the CoronaVac vaccine than in those without a history (p < 0.001)^16^. This discrepancy between studies may be explained by the limited sample size and the use of various types of vaccines.

Nurses made up the largest proportion of healthcare staff in Mali, representing approximately 28% of all participants. In general, healthcare personnel in Mali are more represented by professionals (69%) than physicians^24^; this has also been reported in previous studies conducted in Mali by Somboro et al.^9^ and Maiga AI et al.^23^. All these studies mention that nurses represent the most represented personnel in Mali's health sectors of all healthcare workers. However, no significant differences were detected in terms of the anti-SARS-CoV-2 IgG antibody seroprevalence between the HCWs in different occupations.

We noted that very few cases of comorbidity were reported in this study, which is likely related to the young average age of the participants. High blood pressure was the most frequent comorbidity recorded (3.5%). However, our previous study conducted in Mali revealed that obesity was the most frequent comorbidity, occurring in 7.6% of patients (9).

A number of participants had a history of COVID-19 (8.9%), contrary to the findings of Mog et al. (17) (18.2%) and Somboro et al. (9) (14.6%). Compared to that of the general population, the prevalence of COVID-19 is believed to be greater among healthcare workers, which can be explained by exposure to patients increasing the risk of SARS-CoV-2 spread in this population. The level of SARS-CoV-2 IgG anti-spike antibodies in people previously infected with SARS-CoV-2 was similar to that in people who never experienced COVID-19. It is important to highlight that our previous study reported high SARS-CoV-2 seroprevalence among HCWs prior to vaccination, indicating that they were mostly exposed to SARS-CoV-2, which can explain the similarity between these two groups^9^.

Of the 172 participants, 107 (62.2%) received a two-dose vaccination schedule, and the Astra Zeneca® vaccine was the most widely used. The average time between the 1st and 2nd doses was 32.2± 6.1 days. The percentage of participants who received one dose of vaccination was 37.8%, and Johnson & Johnson® was essentially the only type of vaccine administered once. In addition to these two types of vaccines, the Sinovac® vaccine, Covishield®, and Pfizer® were used in small proportions. The average level of postvaccination SARS-CoV-2 antibodies was significantly greater in participants who received two doses of vaccine than in those who received one dose (33.7 index vs. 29.1 index; p=0.02). Self et al. also reported that the efficacy of the Moderna and Pfizer mRNA vaccines was greater than that of the Janssen vaccine^26^. Real-life efficacy data indicate very good efficacy of COVID-19 mRNA and viral vector vaccines from the first dose, including in people with comorbidities and elderly people^27^.

The rate of effectiveness varies between vaccines and the number of doses administered. Indeed, it is estimated at 95% for the Pfizer and Moderna mRNA vaccines^28^, 64.1%, and 70.4% after one and two doses of AstraZeneca, respectively, and 66% for the Johnson & Johnson vaccine^29^-^32^. The ideal COVID-19 vaccination schedule remains unknown^33^, and it is thus far difficult to reliably estimate the time of protection offered by a single or double dose of vaccination^16^.

In general, the postvaccination immune response decreases with increasing vaccination time. For COVID-19 vaccines, neutralizing antibody levels decrease over time for both mRNA vaccines and viral vector vaccines such as Johnson & Johnson®^33^. In our study, the average level of postvaccine antibodies in participants who received their 1st dose of vaccine for more than 6 months was significantly greater than that in participants who received their 1st dose for less than 6 months (33.1 index vs. 29.8 index) (p=0.023). In the literature, several studies have reported the persistence of circulating antibodies 6 to 9 months after the first series of vaccines and have reported that antibody levels decrease from the peak but are detectable for spike (S) IgG and receptor binding domain (DLR) IgG and neutralizing antibodies^34^. Several types of COVID-19 vaccines were used in our study depending on their availability and often interchangeably (heterologous vaccinations), so it is difficult to directly compare the decrease in immunity between these vaccines over time.

The specific relationship between the postvaccine immune response and protection against severe acute respiratory syndrome coronavirus 2 (SARS-CoV-2) infection still needs to be fully understood, and additional data are needed. However, several consistent analyses have shown that a booster dose is useful for enhancing the humoral response in immunocompromised or cancer patients who do not have detectable antibodies or low antibody levels and even in healthy volunteers^33^.

## Conclusion

A significant portion of COVID-19-vaccinated healthcare professionals exhibited a robust immune response, as evidenced by the vigorous generation of antibodies against the SARS-CoV-2 IgG spike protein, which occurred in 98.2% of patients. Individuals who received a two-dose regimen demonstrated elevated SARS-CoV-2 IgG spike protein levels compared to their single-dose counterparts. This finding accentuates the potential benefits of booster shots, warranting further investigation into our specific population's optimal dosage and frequency.

## Figures and Tables

**Table 2:** Types of vaccines used at the first dose

Types of vaccines	Number(n)	Percentage (%)
**Astra Zeneca ^®^**	90	52.3
**Johnson & Johnson ^®^**	65	37.8
**Covishield ^®^**	10	5.8
**Sinovac ^®^**	6	3.5
**Pfizer ^®^**	1	0.6
**Total**	172	100

**Table 3:** Types of Vaccines at the Second Dose

Type of vaccines	Number (n)	Percentage (%)
**Astra Zeneca®**	90	84.1
**Covishield®**	10	9.3
**Sinovac®**	6	5.6
**Pfizer®**	1	0.9
**Total**	107	100

**Table 5:** Induced SARS-CoV-2 IgG Antibody Levels

IgG antibody levels (Index values)	Sero-Prevalence	Number(n)	Percentage (%)
**0 - 2.9**	Sero-negative	2	1.2
**3 – 4.9**	Intermediate	1	0.6
**≥ 5**	Sero-positive	169	98.2
**Total**		172	100.0

## References

[1] Yu M, Xu D, Lan L, Tu M, Liao R, Cai S (2020). Thin-Section Chest CT Imaging of COVID-19 Pneumonia: A Comparison Between Patients with Mild and Severe Disease. Radiol Cardiothorac Imaging.

[2] Sohrabi C, Alsafi Z, O'Neill N, Khan M, Kerwan A, Al-Jabir A (2020). World Health Organization declares global emergency: A review of the 2019 novel coronavirus (COVID-19). Int J Surg Lond Engl.

[3] Coulibaly S (2021). Organ dysfunction during SARS-CoV-2 related respiratory infection in Mali.

[4] Huang C (2021). Pathogenesis of Coronaviruses Through Human Monocytes and Tissue Macrophages. Viral Immunol.

[5] Fehr AR, Perlman S (2015). Coronaviruses: An Overview of Their Replication and Pathogenesis. Coronaviruses.

[6] Rothan HA, Byrareddy SN (2020). The epidemiology and pathogenesis of coronavirus disease (COVID-19) outbreak. J Autoimmun.

[7] Jin Y, Yang H, Ji W, Wu W, Chen S, Zhang W (2020). Virology, Epidemiology, Pathogenesis, and Control of COVID-19. Viruses.

[8] SITREP (2022). Rapport de situation COVID 19 au Mali numéro 226.

[9] Somboro AM, Cissoko Y, Camara I, Kodio O, Tolofoudie M, Dembele E (2022). High SARS-CoV-2 Seroprevalence among Healthcare Workers in Bamako, Mali. Viruses.

[10] Jamai Amir I, Lebar Z, Yahyaoui G, Mahmoud M (2020). Covid-19: virologie, épidémiologie et diagnostic biologique. Option/Bio.

[11] Bonny V, Maillard A, Mousseaux C, Plaçais L, Richier Q (2020). [COVID-19: Pathogenesis of a multifaceted disease]. Rev Med Interne.

[12] WHO Regional Office for Africa (2022). Mali marks a year of vaccination against COVID-19 in the country, with more than 1,000,000 people fully vaccinated.

[13] WHO- Mali Health cluster, Bulletin N°3.

[14] Ben Houmich T, Tali A, Debbagh F, Lamrani Hanchi A, Soraa N (2022). Seroprevalence of SARS-CoV-2 antibodies in vaccinated healthcare workers in Marrakech (Morocco). Int J Immunopathol Pharmacol.

[15] Uysal EB, Gümüş S, Bektöre B, Bozkurt H, Gözalan A (2022). Evaluation of antibody response after COVID-19 vaccination of healthcare workers. J Med Virol.

[16] Bayram A, Demirbakan H, Günel Karadeniz P, Erdoğan M, Koçer I (2021). Quantitation of antibodies against Anti-SARS-CoV-2 spikeprotein after two doses of CoronaVac in healthcare workers. J Med Virol.

[17] Mog C, Bhattacharya S, Baidya S, Das S (2022). Antibody Responses of SARS-CoV-2 Vaccines among Health Care Workers in a Tertiary Care Hospital in Tripura, India: A Cross-Sectional Study. Indian J Community Med Off Publ Indian Assoc Prev Soc Med.

[18] Weinberger B, Grubeck-Loebenstein B (2012). Vaccines for the elderly. Clin Microbiol Infect Off Publ Eur Soc Clin Microbiol Infect Dis.

[19] Wang P, Liu L, Nair MS, Yin MT, Luo Y, Wang Q (2020). SARS-CoV-2 neutralizing antibody responses are more robust in patients with severe disease. Emerg Microbes Infect.

[20] Naaber P, Tserel L, Kangro K, Sepp E, Jürjenson V, Adamson A (2021). Declined antibody responses to COVID-19 mRNA vaccine within first three months [Internet]. medRxiv.

[21] Goodwin K, Viboud C, Simonsen L (2006). Antibody response to influenza vaccination in the elderly: a quantitative review. Vaccine.

[22] Huang YP, Gauthey L, Michel M, Loreto M, Paccaud M, Pechere JC (1992). The relationship between influenza vaccine-induced specific antibody responses and vaccine-induced nonspecific autoantibody responses in healthy older women. J Gerontol.

[23] Maiga AI, Saliou M, Kodio A, Traore AM, Dabo G, Flandre P (2022). High SARS-CoV-2 seroprevalence among health care workers in Bamako referral hospitals: a prospective multisite cross-sectional study (ANRS COV11). Clin Microbiol Infect.

[24] Ministry of Health and Public Hygiene, Mali (2019). Statistical yearbook 2018 of the hospital information system [Internet].

[25] Mukwege D, Byabene AK, Akonkwa EM, Dahma H, Dauby N, Cikwanine Buhendwa JP (2021). High SARS-CoV-2 Seroprevalence in Healthcare Workers in Bukavu, Eastern Democratic Republic of Congo. Am J Trop Med Hyg.

[26] Self WH, Tenforde MW, Rhoads JP, Gaglani M, Ginde AA, Douin DJ (2021). Comparative Effectiveness of Moderna, Pfizer-BioNTech, and Janssen (Johnson & Johnson) Vaccines in Preventing COVID-19 Hospitalizations Among Adults Without Immunocompromising Conditions - United States, March-August 2021. MMWR Morb Mortal Wkly Rep.

[27] Lachâtre M (2022). COVID-19 vaccination: vaccine technologies, real-life efficacy and specificities. Med Mal Infect Form.

[28] Lin DY, Gu Y, Wheeler B, Young H, Holloway S, Sunny SK (2022). Effectiveness of Covid-19 Vaccines over a 9-Month Period in North Carolina. N Engl J Med.

[29] Voysey M, Clemens SAC, Madhi SA, Weckx LY, Folegatti PM, Aley PK (2021). Safety and efficacy of the ChAdOx1 nCoV-19 vaccine (AZD1222) against SARS-CoV-2: an interim analysis of four randomised controlled trials in Brazil, South Africa, and the UK. Lancet Lond Engl.

[30] Vasileiou E, Simpson CR, Shi T, Kerr S, Agrawal U, Akbari A (2021). Interim findings from first-dose mass COVID-19 vaccination roll-out and COVID-19 hospital admissions in Scotland: a national prospective cohort study. The Lancet.

[31] Sadoff J, Gray G, Vandebosch A, Cárdenas V, Shukarev G, Grinsztejn B (2021). Safety and Efficacy of Single-Dose Ad26.COV2.S Vaccine against Covid-19. N Engl J Med.

[32] Patel R, Kaki M, Potluri VS, Kahar P, Khanna D (2022). A comprehensive review of SARS-CoV-2 vaccines: Pfizer, Moderna & Johnson & Johnson. Hum Vaccines Immunother.

[33] Kherabi Y, Fiolet T, Rozencwajg S, Salaün JP, Peiffer-Smadja N (2021). COVID-19 vaccine boosters: What do we know so far?. Anaesth Crit Care Pain Med.

[34] Haveri A, Ekström N, Solastie A, Virta C, Österlund P, Isosaari E (2021). Persistence of neutralizing antibodies a year after SARS-CoV-2 infection in humans. Eur J Immunol.

